# Magnesium, Calcium, Potassium, Sodium, Phosphorus, Selenium, Zinc, and Chromium Levels in Alcohol Use Disorder: A Review

**DOI:** 10.3390/jcm9061901

**Published:** 2020-06-18

**Authors:** Jacek Baj, Wojciech Flieger, Grzegorz Teresiński, Grzegorz Buszewicz, Ryszard Sitarz, Alicja Forma, Kaja Karakuła, Ryszard Maciejewski

**Affiliations:** 1 Chair and Department of Anatomy, Medical University of Lublin, 20-090 Lublin, Poland; ryszard.maciejewski@umlub.pl; 2 Faculty of Medicine, Medical University of Lublin, Aleje Racławickie 1, 20-059 Lublin, Poland; wwoj24@wp.pl; 3 Chair and Department of Forensic Medicine, Medical University of Lublin, 20-090 Lublin, Poland; grzegorzteresinski@umlub.pl (G.T.); g.buszewicz@umlub.pl (G.B.); aforma@onet.pl (A.F.); 4 Chair and 1st Department of Psychiatry, Psychotherapy and Early Intervention, Medical University of Lublin, Gluska Street 1, 20-439 Lublin, Poland; r.sitarz@hotmail.com (R.S.); kaja.karakula@gmail.com (K.K.)

**Keywords:** alcoholism, alcohol use disorder, zinc, chromium, selenium, calcium, potassium, magnesium, phosphorous, sodium

## Abstract

Macronutrients and trace elements are important components of living tissues that have different metabolic properties and functions. Trace elements participate in the regulation of immunity through humoral and cellular mechanisms, nerve conduction, muscle spasms, membrane potential regulation as well as mitochondrial activity and enzymatic reactions. Excessive alcohol consumption disrupts the concentrations of crucial trace elements, also increasing the risk of enhanced oxidative stress and alcohol-related liver diseases. In this review, we present the status of selected macroelements and trace elements in the serum and plasma of people chronically consuming alcohol. Such knowledge helps to understand the mechanisms of chronic alcohol-use disorder and to progress and prevent withdrawal effects, also improving treatment strategies.

## 1. Introduction

Alcohol dependence among adults in the United States is estimated at 14% [1]. Among heavy drinkers, alcoholic liver disease is estimated to develop in 15–30% [2]. In patients who consume excessive amounts of alcohol, various types of disturbances are observed, i.e., electrolyte, acid-base, protein-caloric malnutrition, and vitamin deficiency [3,4,5]. Chronic alcohol abuse patients are malnourished not only because of a diet low in nutrients but also because alcohol impairs the absorption of essential nutrients and elements. In addition, ethanol metabolic pathways produce toxic metabolites (acetaldehyde and free radicals) that lead to cell damage as a result of oxidative stress [6,7].

The clinical picture of the observed disorders depends on the duration and amount of alcohol consumed. Most patients who develop electrolyte imbalance, metabolic acidosis, and hyponatremia are admitted to hospital. However, clinical symptoms of chronic alcohol consumption are also decreased levels of phosphate, magnesium, potassium, sodium and calcium, and other elements in blood plasma [8,9,10]. Electrolyte abnormalities develop as a result of chronic alcohol consumption during acute alcohol intoxication, but they are particularly important during alcohol withdrawal [11,12,13,14]. It turns out that even during alcohol withdrawal, hypokalemia, hypomagnesemia, and hyponatremia are observed [15,16].

Currently, it is believed that the toxic effects of alcohol on organs are mainly associated with the activity of alcohol metabolites, induction of oxidative stress, and translocation of intestinal endotoxins into the bloodstream [17,18,19,20]. These processes lead to cell damage and stimulation of inflammatory reactions releasing a large number of cytokines, among others such as TNF-alpha and IL-6 [21,22]. With continuous alcohol abuse, which stimulates hepatocytes to secrete some interleukins, such as IL-8, activating Kupffer cells by binding to toll-like receptors, other effects are impaired including defective tissue regeneration, hepatocyte necrosis, recruitment of neutrophils and immune cells, and liver fibrosis [23].

**Figure 1:**
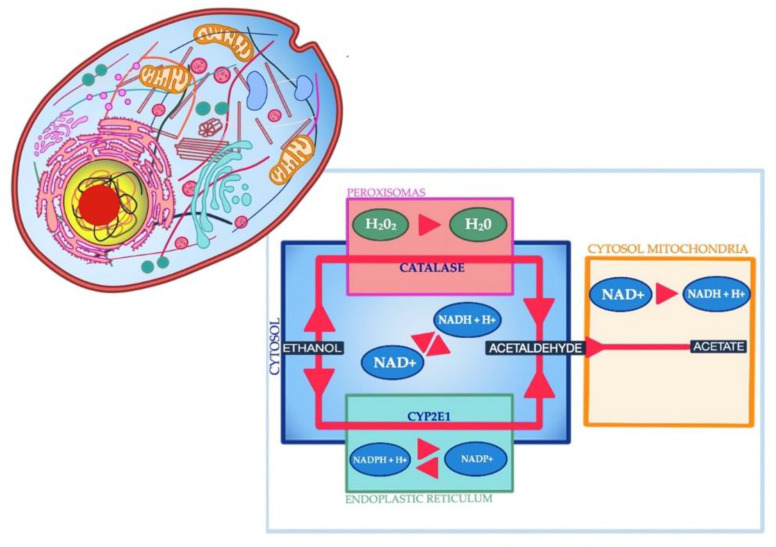
Three pathways of alcohol metabolism.

Alcohol is metabolized in three pathways, involving enzymes such as the alcohol dehydrogenase present in the cytosol, the Microsomal Ethanol Oxidizing System (MEOS), and the catalase (CAT) contained in peroxisomes (Figure 1).

Each of the three pathways is responsible for the production of free radicals [24], which can damage lipids, proteins, carbohydrates, and DNA substrates [25]. However, there are several endogenous enzymes that protect the body against the adverse effects of free radicals, including glutathione peroxidase (GPx), which neutralizes hydrogen peroxide and organic peroxides, glutathione reductase (GR), superoxide dismutase (SOD) and CAT [26]. Cofactors for antioxidant enzymes are trace elements such as selenium, manganese, copper, zinc, and iron. Selenium in the form of selenocysteine is present in the active center of GPx. In turn, manganese, copper, and zinc are components of the enzymes from the superoxide dismutase group (MnSOD, Cu/ZnSOD), which catalyze the reaction of the superoxide anion radical dismutation to hydrogen peroxide and oxygen. Iron is a part of CAT that catalyzes the decomposition of hydrogen peroxide into water and oxygen. Zinc-dependent enzymes, in turn, are alcohol dehydrogenase, RNA polymerases, and fructose 1,6-bisphosphatase, which is allosterically regulated by zinc and could influence gluconeogenesis. Therefore, trace element concentrations have a significant impact on the activity of antioxidant enzymes, and thus, on the modulation of ethanol-induced oxidative stress. Research to date has shown that alcoholism significantly changes macroelement homeostasis, i.e., calcium, phosphorous, magnesium, sodium, potassium, and trace minerals, which, as cofactors for antioxidant enzymes, play a role in the fight against oxidative stress and may affect survival (Table 1).

The purpose of this paper is to present the effects of excessive and chronic alcohol consumption on magnesium, calcium, sodium, potassium, phosphorus, selenium, zinc, and chromium levels, as balanced homeostasis is crucial to prevent the dysfunctions of vital processes and functions. Besides, the authors have presented the mechanisms of the abovementioned alterations in macro- and microelement concentrations, as well as the subsequent side effects and clinical manifestations of such alterations. Further recommendations for future research and treatment options for alcohol use disorder are also evaluated.

## 2. Materials and Methods

Literature from PubMed, Google Scholar, and Web of Science databases was extracted and included original articles as well as review articles (including systematic reviews and meta-analyses) that were published within the time period 1952–2020. The inclusion criterion was the English language; no limits were included for the publication year. Additionally, both human and animal studies were included in the final analysis. The literature was analyzed in terms of changes in chosen macro- and microelement concentrations in cases of prolonged and excessive alcohol intake and alcohol use disorder in particular. The chosen elements were magnesium, calcium, sodium, potassium, phosphorus, selenium, zinc, and chromium, and this inclusion criterion was based on the fact that alterations of the abovementioned elements are highly prevalent among alcohol-dependent patients and usually result in clinical manifestations with a different degree of severity. The search strategy included the following keywords: (alcohol use disorder OR alcohol OR alcoholism) AND (electrolyte OR microelement OR macroelement OR mineral OR phosphorus OR hypophosphatemia OR magnesium OR hypomagnesemia OR calcium OR hypocalcemia OR potassium OR hypokalemia OR sodium OR hyponatremia OR selenium OR zinc OR chromium). In addition to the literature search on PubMed, Google Scholar, and Web of Science databases, the references included in the analyzed papers were also taken into consideration. Finally, 209 articles were assessed as relevant to the topic and included in the review.

## 3. Acid-Base Disturbances

Chronic alcohol users are prone to various acid-base disorders. Most patients most often have mixed disorders (78%) [44]. Alcoholic metabolic acidosis occurring in almost half of patients is manifested by an increased anion gap, which is the result of the accumulation of ketoacids, lactic acid, acetic acid, or indirect loss of bicarbonate through urine [45,46]. Almost a third of patients also have symptoms of respiratory alkalosis [47]. The development of ketoacidosis leads to an increase in the ratio of reduced NADH to oxidized nicotinamide adenine dinucleotide (NAD) and further formation of β-hydroxybutyrate. In turn, the increased ratio of NADH to NAD has an inhibitory effect on hepatic gluconeogenesis, which leads to life-threatening hypoglycemia [48,49].

## 4. Phosphorus Deficit

Acute hypophosphatemia associated with phosphorus deficiency develops by up to 50% in patients within the first 2–3 days after hospitalization due to problems associated with chronic alcohol abuse [50,51]. Phosphorus deficiency is most often caused by an inadequate diet low in phosphates or due to alkalizing drugs, chronic diarrhea, or vomiting that prevent efficient phosphorus absorption. At the same time, despite hypophosphatemia, increased urinary phosphate excretion is observed as a result of renal tubular dysfunction accompanied by glycosuria, amino acid urine, hypomagnesuria, and hypercalciuria [52]. It is believed that the observed disorders are the result of structural changes in the phospholipid bilayer of the renal tubules, a dysfunction of apex-located transporters, and reduced potassium-sodium ATPase activity [53,54]. In addition, increased renal excretion of phosphate in patients with metabolic acidosis results from the fact that phosphate releases from bones and is a direct effect of pH gating in proximal tubules with the NaPi-2a and NaPi-2c co-transporters [55].

Reduced phosphate reabsorption may be the result of increased parathyroid hormone (PTH) levels due to hypocalcemia induced by vitamin D deficiency or magnesium deficiency due to phosphaturia [56]. It has been documented that magnesium deficiency can lead to a decrease in the phosphate content of skeletal muscles and an increase in their excretion in urine. Thus, phosphorus deficits play a large role in the etiology of alcohol myopathy and acute rhabdomyolysis [57,58].

Magnesium deficiency can cause a state of hypoparathyroidism, thus contributing to phosphaturia. The mere normalization of pH in patients with ketoacidosis causes an intracellular phosphate shift, which in turn, leads to hypophosphatemia. This is confirmed by skeletal muscle biopsy studies that show a reduced content of phosphates and magnesium, which are accompanied by increased amounts of sodium, chloride, and calcium [59,60,61]. Hypophosphatasemia is also involved in the development of metabolic acidosis. Intracellular phosphate deficiency interferes with the production of ATP, which in turn, stimulates phosphofructokinase activity, enhancing glycolysis and lactate production. Besides, cellular phosphate deficiency in red blood cells causes tissue ischemia and increases lactic acid production.

## 5. Imbalances in Magnesium and Calcium Levels in Plasma

Almost a third of chronic alcohol users have hypomagnesemia [62]. Reduced magnesium content in the body is the result of insufficient consumption of products rich in magnesium, as well as impaired absorption due to chronic diarrhea associated with, among others, impaired digestion or absorption of fats, which are excreted in the form of fatty acid–magnesium complexes (steatorrhea). Magnesium concentration should be measured in serum rather than in the plasma because the anticoagulant used for plasma collection may be contaminated with magnesium or may affect the test procedure. For example, citrate binds not only calcium but also magnesium and affects the measurement result by fluorometric and colorimetric methods. It is also important to avoid hemolysis because the magnesium concentration in red blood cells is about three times higher than the serum concentration and it has been estimated that the serum magnesium concentration will increase by 0.05 mM/L for each g/L hemoglobin produced by hemolysis. Serum total magnesium may also be affected by high bilirubin, lipemic serum, and high phosphate [63]. Magnesium deficiencies are closely related to phosphate deficiencies (Table 2).

Magnesium deficiency leads to the kidneys losing phosphate, while phosphate deficiency is associated with kidneys losing magnesium, resulting in reduced magnesium and ATP content in skeletal muscle.

The development of hypomagnesemia inhibits the release and induces peripheral resistance to parathyroid hormone, leading to hypocalcemia [94]. To reverse this process, the restoring of the physiological magnesium levels is inherent. Vitamin D deficiency is also an important risk factor for hypocalcemia, like alcohol-related steatorrhea or rhabdomyolysis.

Calcium plays an important role in the proper functioning of the body. Most calcium (99%) is found in teeth and bones, which can be considered as its reservoir [95,96]. Only 1% of total calcium is found in the serum. Adequate calcium levels in the brain activate a wide range of Ca^2+^-dependent processes in neurons, including neurotransmitter release, gene transcription, activation of Ca^2+^-dependent enzymes, and activation of some K^+^ and chloride channels [97,98].

The serum calcium content is regulated by calcitonin, vitamin D, and parathyroid hormone (PTH) [99,100]. According to research, acute alcohol consumption leads to an increase in calcitonin plasma levels, while chronic alcohol consumption and detoxification have varying effects [101,102]. Chronic alcohol consumption is known to lead to vitamin D deficiency, reduced intestinal calcium absorption (by up to 80%), which in turn, causes osteoporosis and osteopenia [103,104]. Reduced plasma calcium levels have been demonstrated in alcohol-dependent patients with and without cirrhosis [105]. Chronic alcohol consumption leads to an increase in phosphoinositide-dependent cytosolic calcium levels, disturbing mitochondrial Ca^2+^ levels and energy metabolism. In addition, Laitinen et al. [106] presented that defective parathyroid function was found to respond to hypocalcemic stimuli in alcohol-dependent patients during intoxication.

In 1952, O’Brien showed that intravenous calcium administration reduces withdrawal symptoms in alcohol-dependent patients [107]. In an animal model, it was confirmed that calcium, which is a component of the drug that reduces cravings for alcohol addicts (acamprosate calcium acetyl-homotaurine), is involved in preventing alcohol cravings in addicted patients [108,109]. This conclusion has not been confirmed by other authors [110]. Schuster et al. in 2016 conducted a plasma calcium concentration test in alcohol-dependent patients [111]. The psychometric dimension of hunger was studied using the OCDS, ADS, and ADS-HR scales. It was found, among others, the existence of a negative correlation between plasma calcium levels and alcohol cravings, a negative correlation of plasma calcium levels with alcohol concentration in the exhaled air, decreased calcitonin concentration in the group of high-risk alcoholics, and decreased vitamin D plasma concentration. The authors suggest the usefulness of calcium supplementation in reducing cravings and relapse in people addicted to alcohol. Recent studies reveal the mechanisms of disorders seen in alcoholics during abstinence. As demonstrated, voltage-sensitive calcium channels Ca^2+^ (CaV) in the brain are an important target for alcohol. Alcohol-induced changes in Ca^2+^ signaling may interfere with neuronal homeostasis, Ca^2+^-mediated gene transcription, and neural circuit function, leading to various neurological symptoms and neuropsychiatric disorders, including alcohol withdrawal convulsions and alcoholism [100].

## 6. Plasma Potassium Deficit

Hypokalemia occurs in almost 50% of patients with chronic alcohol consumption disorder [112]. Plasma potassium concentrations and incidents of hypokalemia due to alcohol intoxication might differ depending on the age of patients or coexistence of concomitant diseases. Potassium deficiency results from an inadequate diet and loss of potassium through the gastrointestinal tract due to malnutrition, diarrhea, vomiting, and increased loss of urine. Further to this, glucose infusion induces potassium shift from extracellular to intracellular compartments. The most serious symptoms of hypokalemia include myopathy, rhabdomyolysis, and cardiac effects—from asymptomatic electrocardiographic changes to potentially life-threatening cardiac arrhythmias. Central pontine myelinolysis might also be a severe consequence of hypokalemia among alcohol-dependent patients [113,114]. The above symptoms are the result of an increase in mineralocorticoid levels and an increased supply of sodium to the distal nephron. Under normal circumstances, intracellular magnesium blocks potassium channels (ROMK) in the apical membrane of the distal nephron and limits potassium losses [115,116]. Coexisting magnesium deficiency in serum causes a decrease in its level inside the cell, which results in the prevention of inhibition of ROMK channels and is responsible for potassium losses [112]. Stimulation of β2-adrenergic receptors in skeletal muscle due to respiratory alkalosis contributes to the development of hypokalemia. It is recommended to measure plasma potassium, sodium, glucose, and lactate levels among patients with alcohol intoxication [117]. Potassium and platelet levels are crucial in predicting the severity of alcohol withdrawal [118,119]. Hypokalemia along with hypochloremia are associated with a higher risk of delirium tremens. Furthermore, elevated levels of plasma catecholamines, especially during the alcohol withdrawal period, induce intracellular shift of potassium and magnesium, which explains the relationship between hypokalemia, hypomagnesemia, and further delirium tremens [15].

## 7. Sodium Deficit

Hyponatremia is the most common electrolyte disorder seen in people consuming excessive amounts of alcohol. Hyponatremia is diagnosed when the sodium plasma concentration is below 135 mM/L. Sodium is responsible for maintaining basic vital functions. It is involved in maintaining water and electrolyte balance [120], acid-base [121], osmotic pressure [122], transport of amino acids, carbohydrates and vitamins [123] serotonin (SERT), dopamine, noradrenaline, GABA [124,125], excitability neuromuscular [126], and conduction of nerve impulses [127]. Physiological sodium levels in the plasma range from 135 to 145 mM/L (Table 3).

Sodium presence has a large impact on plasma osmolality, since salts of sodium (chloride and bicarbonate) along with urea and nonelectrolyte glucose are the most inherent osmoles of plasma. Many studies describe reduced plasma sodium levels in alcohol-dependent patients compared to the control group [133,134,135,136,137].

There are three types of hyponatremia: mild (130–134 mM/L), moderate (120–129 mM/L), and severe (less than 120 mM/L); furthermore, hyponatremia can be classified as acute or chronic [138,139]. Clinical manifestations of hyponatremia depend on its severity and duration. Acute hyponatremia, for instance, is characterized by the onset of neurologic symptoms (such as seizures, impaired mental status, coma, or death) [140]. Further to this, central pontine myelinolysis might appear, which has an impact on the risk of complications and worsening prognosis of comorbidities, as well as on mental condition and quality of life, related to health. Contrarily, patients with chronic hyponatremia can be asymptomatic. Mild hyponatremia is primarily characterized by gastrointestinal symptoms (nausea, vomiting, loss of appetite) and sometimes by neurologic impairments [141]. Moderate and severe cases of hyponatremia are characterized by a higher probability of deterioration of neurologic manifestations.

Ordak et al. [142] studied the effect of hyponatremia in alcohol-dependent patients on the physical and mental state of patients. Based on verification using the BSI clinical symptom scale, Barratt impulsivity scale, SDQ-7 sleep disorder questionnaire, SF-36 quality of life questionnaire, list of five NEO-FFI factors, and MAST (Michigan Alcoholism Screening Test), the researchers confirmed that the lower the sodium concentration in plasma was, the higher was impulsiveness and neuroticism, quality of life, and mental aspect.

High alcohol consumption induces diuresis by increasing the level of vasopressin, which predisposes patients to dehydration and hypernatremia [143,144]. This is a common disorder that occurs in 17% of patients chronically consuming alcohol [145]. However, this effect may not be observed in the case of prolonged alcohol consumption. In such patients, the level of vasopressin increases, which results in an increase in urine osmolality and a decrease in free water clearance.

However, there is hyponatremia independent of vasopressin, which occurs with a long-term beer-based diet—“beer offspring”. “Beer Potomania” is a unique hyponatremia syndrome that was first described in 1972 [146,147,148,149]. The low content of nutrients in beer and the suppressing effect of alcohol on proteolysis cause secondary dilution hyponatremia, which leads to reduced removal of excess fluid from the body. Progeny includes severe hyponatremia (plasma sodium concentration, <110 mmol/L), hypokalemia, low blood urea nitrogen (indicating low protein intake), and maximum diluted urine (<100 mOsm/kg of water) [150].

The impact of hyponatremia on brain metabolism has been the subject of in-depth studies [151,152,153,154]. It turns out that hyponatremia has a complex, nonlinear relationship with the brain Glx metabolites (glutamate + glutamine), cognitive ability, and generally understood, health-related quality of life (HRQOL) [151,155]. Magnetic resonance spectroscopy (MRS) studies have shown that in a state of edema caused by hyponatremia, a decrease in the content of choline and myo-inositol (mI) in the brain is observed [156,157]. The cerebral metabolic profile of patients with hyponatremia is characterized by a low ratio of mI/Cr (creatinine) and Glx/Cr, as a result of which there may be a high risk of developing cerebral edema following a hyperammonemic stimulus. The relationship between serum sodium and Glx/Cr was nonlinear and linear with negative mI/Cr. In addition, mI/Cr ratios were significantly correlated with weak HRQOL in physical and psychosocial dimensions. At the same time, it was shown that the concentration of mI/Cr increases after the correction of hyponatremia. It should be remembered, however, that if hyponatremia is corrected too quickly, myelinolysis of the middle bridge may occur [158].

Under these circumstances, myelin glial cells are exposed to osmotic stress. However, this condition should be taken into account in any patient with alcohol dependence syndrome, even if there is no sodium flow, as these patients have a direct toxic effect of alcohol through chronic osmotic stress, which may play an important role in the pathophysiology of the observed changes.

## 8. Selenium Deficit

Selenium (Se) is an essential trace element, important for human health due to its anti-inflammatory, chemopreventive, and antioxidant activity realized through various selenoproteins [159]. It is an essential component of glutathione peroxidase (GPx) and phospholipid-hydroperoxide-glutathione peroxidase. It is believed that selenium counteracts the effects of oxidative stress mainly by GPx [159]. Normal serum selenium concentration is 50–120 μg/L. Low serum Se levels have been confirmed many times in patients with hepatic complications as well as in alcohol-dependent patients [151,160,161,162,163].

Rua et al. studied the effect of chronic ethanol consumption on oxidative balance and selenium (Se) levels in patients chronically consuming alcohol with or without concomitant liver disease [35]. They found that serum Se levels were lower in alcoholic patients and patients with liver disease, and particularly lower in the group of alcoholic patients with concomitant liver disease. These values were correlated with the profile of antioxidative enzymes, i.e., glutathione peroxidase (GPx), selenoproteins, glutathione reductase (GR), and superoxide dismutase (SOD). Heavy alcohol consumption and related Se deficiency, primarily lower serum GPx3 and liver GPx1 activities, as well as hepatic GPx4 and GPx1 expressions [164].

Thus, both chronic alcohol abuse and liver disease are associated with a decrease in Se levels. Therefore, there is controversy regarding the evaluation of these results. By Bergheim et al. [165] and Ojeda et al. [166], a decrease in serum and liver levels of Se can cause alcohol alone. In turn, the Se deficit further causes a type of hepatocyte necrosis similar to that observed in alcoholic patients, as demonstrated in animal studies by Simonoff and Simonoff [167]. On the other hand, it is known that liver diseases [163] also contribute to low levels of Se by disrupting the synthesis of selenoproteins involved in the transport of selenium in the serum [168]. Zhu et al. [169] and Rua et al. [35] have shown, however, that in non-alcoholic liver damage, oxidative balance differs significantly from that of chronic alcohol exposure. Alcohol consumption decreases antioxidant defense in both liver tissue and blood. These changes in the activity of antioxidant enzymes are associated with an increase in protein and lipid oxidation in alcohol patients, confirmed by MDA levels, which are particularly higher in alcohol patients [140,170,171,172,173]. The authors proposed the ratio of Se to malonic aldehyde, which is an indicator of oxidative damage (Se/MDA), as an effective diagnostic tool for assessing liver damage and its etiology. The above indicator is 37.5% lower in non-alcoholic liver disease, 65.7% in alcoholics without liver disease, and 84% in alcoholics with liver disease. Alcohol-dependent patients with concomitant liver diseases present even more decreased Se levels compared to those without coexisting diseases [43]. Selenium causes a decrease in liver fat amount and hepatocyte ballooning among patients with alcohol-related liver steatosis [174]. Considering the fact that alcohol is a pro-oxidant and Se is a mineral with antioxidant capacity, of which reserves may be depleted in patients after chronic alcohol exposure, Se supplementation may be considered as a possible antioxidant therapy, slowing down the progression of secondary alcohol diseases. A simultaneous Se and Mg supplementation provide enhanced antioxidant defense, being more effective in the prevention of oxidative stress, as well as normalization of liver functions and lipid parameters. Furthermore, the release of pro-inflammatory cytokines (TNF-α and IL-1β) is inhibited by an anti-inflammatory cytokine, IL-10, highly excreted during Se supplementation [175].

## 9. Zinc Concentration Disturbance

Zinc (Zn) is effectively absorbed from food. It should be noted, however, that while animal proteins improve its absorption, due to the formation of more soluble complexes with low molecular weight ligands like histidine, methionine, the phytates (myo-inositol hexaphosphate, pentaphosphate) present in plant products, they irreversibly bind zinc in the intestinal lumen, which negatively affects its absorption. Zn has many important physiological functions. Zn is a structural component of the antioxidant enzyme, Cu/Zn-SOD, also acts as a stabilizer of biological membranes, and is an important factor in controlling the transcription of genes responsible for cell proliferation and differentiation as well as intracellular signaling [176,177]. Zn is an essential structural component of about 2500 Zn-finger proteins, which constitute about 8% of the human genome [178,179]. Zn is also necessary for proper functioning of the immune system [180,181,182].

Chronic heavy alcohol consumption disturbs zinc homeostasis, decreasing serum Zn concentration by over 25% compared to healthy individuals, also affecting plasma, erythrocyte, and hepatic concentrations. Experimental studies proved that alcohol-induced decreased hepatic Zn levels are due to the alterations of hepatic zinc transporters’ downregulation of Zip5 and Zip14, and upregulation of Zip7 with Znt7 [89,183,184]. Ethanol and its metabolites stimulate Kupffer cells to release interleukins, which is further implicated in lowered plasma Zn levels. Zn deficiency is observed in approximately 30–50% of alcoholics, being the most frequent nutritional manifestation in alcohol-related liver diseases [185]. Zn deficiency in alcoholics is of a multifactorial origin, among which alcohol-related reduction in exocrine functions of the pancreas, as well as reduction in numerous ligands, are of major importance. Furthermore, Zn metabolism is directly affected by alcohol, as well as because of homeostatic alterations due to the hepatic failure itself. Chronic alcohol exposure impairs Zn homeostasis mainly due to reactive oxygen species, lipid peroxidation, and acetaldehyde. Besides, Zn deficiency decreases the activity of alcohol dehydrogenase, eventually slowing down ethanol elimination. Several studies proved that the maintenance of a balanced diet during alcohol dependence prevents altered Zn distribution [186]. There are speculations that low levels of Zn cause neuronal damage and brain dysfunctions, lymphopenia, reduced ability to respond, and susceptibility to ethanol withdrawal attacks. There is a relationship between decreased Zn and subsequent lowered vitamin A levels, and the severity of hepatic lesions. Zn supplementation inhibits oxidative stress and a following oxidative liver injury, primarily due to the inhibition of the metabolic shift from aldehyde dehydrogenase to CYP2E1 [2,187]. It also decreases alanine aminotransferase levels, which are elevated during chronic alcohol consumption. Therefore, some authors recommend daily zinc supplementation for the treatment of alcohol withdrawal symptoms, as well as alcohol-related brain dysfunctions [188,189,190]. Zima et al. [24] and Menzano and Carlen [188] described the effect of reduced serum zinc in alcoholics without liver damage on the immune system disorder and brain dysfunction.

Alcohol-modulated zinc transporter modulation has recently been shown to be one of the causes of decreased Zn levels in the lungs, liver, intestines, and brain [89]. Apart from the direct effects of alcohol on the intestinal epithelial barrier, Zn deficiency might further sensitize epithelial cells to alcohol, exaggerating symptoms, and worsening the clinical outcome of alcohol-dependent patients [191]. Deficiency of Zn in the intestine causes an increase in intestinal permeability, which ultimately leads to endotoxemia and systemic inflammation. Similarly, a deficiency of Zn in lung epithelium and alveolar macrophages reduces the function of the pulmonary barrier, causing respiratory distress syndrome. Finally, impaired bowel and liver function causes the accumulation of neurotoxic metabolites, which can play a significant role in alcoholic brain damage. There is also a hypothesis that ethanol-induced Zn deficiency may interfere with neurotransmission.

## 10. Chromium Deficit

Two levels of chromium oxidation are considered to be biologically relevant i.e., compounds containing chromium (6+), which are mutagenic and carcinogenic, and chromium (3+), which was a recognized essential element for mammals involved in carbohydrate, fat, and protein metabolism [93,192]. However, in the last two decades, its status has been questioned many times. In 2014, the European Food Safety Authority found no convincing evidence that chromium is an essential element [93,193].

The content of chromium in the liver is estimated at 1 μmol/kg BW, while the total content of this element is about 100 μmol/kg BW. Chromium is absorbed by passive diffusion in only about 1%. Chromium remains in the bloodstream associated with protein-transferrin and α-globulin, from where it is delivered to tissues by endocytosis [93,194]. Data on chromium content standards vary widely. In 2008, Walter et al. compared the distribution of Cr ions in whole blood, plasma, serum, and erythrocytes [195]. They concluded that most of the Cr was found in the extracellular space of the blood. Serum and plasma concentrations were highest and lowest in erythrocytes. The authors recommend using serum or plasma fractions for comparative testing of Cr levels. In the same year, De Smet et al. considered Cr > 17 μg/L as a manifestation of metallosis [196].

Chromium in its trivalent (Cr^3+^) chelated form (Cr-nicotinic acid-glutathione) is considered a glucose tolerance factor (GTF) and it has been postulated that it is involved in regulating the metabolism of not only carbohydrates but also lipids and potentially, protein. The best-defined task of Cr is to act as a cofactor for a biologically active molecule that enhances the effect of insulin on target tissues. Its role in the treatment of diabetes in animals was described in the 1950s, but the impact of Cr on the etiology of diabetes in humans is still controversial. Many authors confirm that patients with chromium deficiency develop severe diabetes that does not respond well to insulin, which can be corrected by chromium supplementation. It has been found that such long-term chromium deficiency develops in patients dependent on parenteral nutrition. Additionally, in people with type 2 diabetes, Cr supplementation may improve glucose metabolism [197,198,199]. A similar effect was demonstrated in patients with severe insulin resistance who were given intravenous chromium (III) [200,201]. Based on the results of Jain et al. (2012), it was stated that Cr supplementation has the potential as an adjunct therapy for patients suffering from type 2 diabetes [202].

Studies on the effects of Cr supplementation on carbohydrate and lipid metabolism in healthy individuals are often contradictory [203]. Some authors show in their studies that there are no beneficial effects in healthy people [204,205], emphasizing that Cr therapy not only does not affect insulin sensitivity but paradoxically reduces it [206]. There are also several publications describing the positive role of Cr in reducing lipid content and body weight in obese people and animals [207,208,209]. Specifically, an interaction between ethanol and chromium alters biochemical parameters such as liver total triglycerides, liver glycogen, succinate dehydrogenase, or liver glutathione.

Hypoglycemia also accompanies alcoholism, which can be a manifestation of acute hypochromism. In the medical literature, hypoglycemia in alcoholics has not been identified as a consequence of chromium deficiency, although insulin use, in this case, is not always effective. Most authors agree that chronic alcohol consumption is a potential risk factor for type 2 diabetes (T2DM), which causes insulin resistance and pancreatic β cell dysfunction.

## 11. Conclusions

Chronic alcohol consumption alters essential micro- and macronutrient levels leading to a significant number of metabolic disorders, among which alcohol-related liver disease and pancreatitis are of the highest prevalence. The severity of alcoholic liver disease varies from simple steatosis to liver cancer. Patients with alcoholic liver disease should be encouraged to discontinue alcohol consumption. Alcohol administration is associated with increased damage due to oxidative stress and significantly lowered trace elements and antioxidant enzyme levels. There is a multitude of causes of the abovementioned imbalances, including inappropriate nutritional status, alcohol-related gastrointestinal disorders, diarrhea, vomiting, or excessive urination. Electrolyte and trace element imbalances are observed both during alcohol administration and alcohol withdrawal because of the defective functioning of renal tubules impaired by alcohol. The most common distortions include hypophosphatemia, hypomagnesemia, hypokalemia, hypocalcemia, and hyponatremia, as well as decreased levels of selenium, chromium, and zinc. A proper understanding of the pathomechanisms of electrolyte imbalances during alcohol administration and withdrawal provides the most effective treatment strategies for such patients, significantly increasing their clinical outcome. The treatment of electrolyte disturbances in alcohol use disorder depends on the degree of deficiency and subsequent clinical manifestations. It is crucial to provide either oral or intravenous supplementation of relevant electrolytes in order to reach their proper physiological ranges. Besides, close monitoring of patients is required, as electrolyte disturbances might persist even during the alcohol withdrawal period or after the alleged initial correction of electrolyte concentrations. Further evaluation of possible treatment strategies and their improvements is crucial to minimize the potentially toxic side effects of these therapies as well as to shorten the duration of the recovery time.

## Figures and Tables

**Table 1:** Minerals deficiency in patients of alcohol abuse.

Sample Source	Concomitant Diseases	Patients	Determined Element	Results of Other Measured Parameters/Treatment	Ref.
serum	without	108 (male and female)	40% of subjects had low Zn levels	-the low serum zinc group had the higher AST: ALT ratio, the lower albumin levels, and the higher CRP, lower bilirubin levels.	[27]
serum	without	7 patients	low Zn levels	-albumin, transferrin, and prealbumin were depressed in zinc-deficient patients.	[28]
serum	HIV infection	7 (AD + HIV), 7 (AD)	lower Zn in AD + HIV patients.	-AD + HIV: elevation in LA, ALA, ALT.	[29]
erythrocytes in blood plasma	without	100 AD patients, 50 the control group	AD had reduced level of Zn	-zinc in erythrocytes/copper in blood plasma ratio differs from alcohol-dependent patients in comparison to the control group.	[30]
serum	gastric bypass surgery	a case report (*n* = 1)	Zn deficiency	-hypomagnesemia (0.9 mg/dL [1.6–2.3 mg/dL]) and hypophosphatemia (2.2 mg/dL [2.5–4.5 mg/dL]) were observed, -Hb 8.7 g/dL (normal 13.5–17.5 g/dL), and liver functions consistent with alcoholism (AST 139 U/L [8–20 U/L], ALT 52 U/L [8–20 U/L], alkaline phosphate 196 U/L [20–70 U/L], and total bilirubin 2.9 mg/dL [0.1–1.0 mg/dL]). INR was 1.3.	[31]
serum, alveolar macrophages	without	alcoholics (*n* = 17) and control (*n* = 17)	AD had normal serum Zn	-AD patients had decreased alveolar macrophage intracellular zinc levels; bacterial phagocytosis; and expression of granulocyte-macrophage colony–stimulating factor receptor β subunit.	[32]
serum	pellagra	2 cases	Zn deficiency	-supplementation with niacin and zinc.	[33]
serum	chronic pancreatitis	23 males and 12 females, with a disease, 14 controls (6 males and 8 females)	Zn level was not different	-hemoglobin (130 ± 16 vs. control 143 ± 15 g/L), vitamin E (8 ± 5 vs. 16 ± 9 mg/L), vitamin A (30 ± 11 vs. 49 ± 12 μg/dL), selenium (54 ± 20 vs. 87 ± 11 μg/L), and plasma glutathione peroxidase (903 ± 313 vs. 1326 ± 168 units/L) were significantly lower in AD patients than in controls (*p* < 0.05), -white blood cell count, C-reactive protein, and plasma copper levels were significantly higher in patients than in controls, -cholesterol, triglycerides, iron, ferritin, total proteins, zinc, and malondialdehyde were not different. Vitamin E was lower in patients with steatorrhea, while vitamin A was lower in patients with concomitant diabetes mellitus.	[34]
serum	liver disease	alcoholics without liver disease, alcoholics with liver disease, and non-alcoholics with liver disease; and healthy volunteers	Se levels were lower in AD	-lower the activity of GPx, the antioxidant activities of GR, SOD in the non-healthy groups, -higher concentrations of PC, MDA.	[35]
serum	alcoholic liver disease	AD patients and controls	Se was more depressed in subjects with liver disease	-lower vitamin E levels in AD patients, -no changes in serum GPx activity, -higher activity of lipid peroxides, and transaminase (AST).	[36]
plasma, whole blood, and red blood cell	without	30 AD patients (group I) and 20 controls (group II).	Lower Se levels -plasma 0.065 μg/mL (I)vs. 0.100 μg/mL (II)	-percentage of ideal body weight, midarm muscle circumference, serum albumin, and total lymphocyte count revealed no differences, -elevated AST and/or alkaline phosphatase	[37]
maternal (1 and 3 trimesters) and umbilical cord blood	without	A total of 30 pregnant women (15 women consuming alcohol and 15 controls)	AD patients had increased Ca and Na in the first trimester of pregnancy. In the third trimester, Co levels increased.	-maternal alcohol consumption results in fetal Co and Mn deficiency, -in the third trimester in women consuming alcohol, we detected a close association between maternal whole blood and cord blood levels for Ca, Cd, and Pb.	[38]
serum	non-traumatic rhabdomyolysis	a case report (*n* = 1)	hypokalemic, Mg deficiency.	-abrupt withdrawal may exacerbate hypokalemia and hypomagnesemia increasing the risk of rhabdomyolysis, -other measured parameters: potassium of 1.4 mmol/L; magnesium 0.40 mmol/L; phosphate 1.40 mmol/L; adjusted calcium 1.87 mmol/L and creatine kinase 6421 U/L.	[39]
serum	tetany	2 alcoholic patients, (a woman aged 64 and a man aged 69 years)	low plasma levels of Ca, Mg, and K.	-hypomagnesemia leads to suppression of parathyroid-hormone secretion, parathyroid-hormone resistance, and vitamin-D suppression, resulting in hypocalcemia, -hypomagnesemia causes kaliuresis leading to hypokalemia, -supplementation with magnesium is crucial in the treatment of this combined electrolyte disorder.	[40]
blood samples	without	58 AD patients (17 females and 41 males)	Zn and Se did not differ from reference values	-alcoholics show a significant increase in blood oxidative stress and Pb and decrease in thiamine, -T/TDP was lower than controls (*p* < 0.005), -ROMs were higher than the healthy population only in female abusers.	[41]
blood samples	chronic alcoholic pancreatitis	chronic alcoholic pancreatitis *n* = 12, alcoholics without visceral disease *n* = 16, and controls *n* = 20	Mg was depleted,	-alcoholic populations suffered from reduced lean body mass (*p* = 0.001), with well-maintained body fat, -depletion of vitamin D and B12, -LDL and total cholesterol were increased in alcoholics without pancreatitis (*p* = 0.04), but not in those with visceral damage, -C-reactive protein and serum amyloid A correlated with the duration of excessive drinking (*p* = 0.01).	[42]
serum	pancreatitis alcoholic liver disease, liver cirrhosis	76 AD patients (ten women), 16 the control group (three women)	Se was lower in AD patients, with the highest mortality.	-patients with liver cirrhosis showed significantly lower Se values than those without cirrhosis -no relation was observed between Se and alcohol intake, -prothrombin activity- as a marker of liver function-shows independent prognostic value.	[43]

AST/ALP—aspartate aminotransferase/alanine aminotransferase, CRP—C-reactive protein, LA—linoleic acid, ALA—alpha-linoleic acid, ALT—alanine aminotransferase, AD—alcohol-addicted patients, GPx—glutathione peroxidase, GR—glutathione reductase, SOD—superoxide dismutase, PC—protein carbonyl, MDA— malondialdehyde, T—thiamine, TDP—diphosphate, ROMs—reactive oxygen metabolites, LDL—low-density lipoprotein, INR—international normalized ratio.

**Table 2:** Clinical features of minerals deficiency (hypocalcemia, hypomagnesemia, hypophosphatemia, hyponatremia, and hypokalemia).

Element	Clinical Manifestations Associated with Deficiency
Magnesium (Mg)	Electrolyte disturbance: hypokalemia (renal potassium wasting, decreased intracellular potassium), hypocalcemia [62]
	Neuromuscular and central nervous system: positive Chvostek and Trousseau signs, spontaneous carpal-pedal spasm, seizures, vertigo, ataxia, nystagmus, athetoid, and choreiform movements, muscular: weakness, tremor, fasciculation, and wasting [64,65]
	Psychiatric: depression, psychosis [65,66]
	Cardiovascular: cardiac arrhythmia, (electrocardiographic: prolonged PR and QT intervals, U waves, atrial tachycardia, premature contractions, fibrillation, hypertension, junctional arrhythmias, ventricular premature contractions, tachycardia, fibrillation, sensitivity to digitalis intoxication), myocardial ischemia/infarction, hypertension, atherosclerotic vascular disease, preeclampsia, atrial tachycardias, fibrillation, supraventricular arrhythmias, ventricular arrhythmias, torsade de pointes, digoxin sensitivity [67]
	Miscellaneous: migraine, asthma, chronic fatigue syndrome, athletic performance [68]
	Bone and mineral metabolism: hypocalcemia (impaired parathyroid hormone secretion, renal and skeletal resistance to parathyroid hormone, resistance to vitamin D), osteoporosis [67]
	Complications of magnesium deficiency: altered glucose homeostasis, atherosclerotic vascular disease, hypertension, myocardial infarction [65]
Calcium (Ca)	Cardiovascular: the characteristic electrocardiographic finding in hypocalcemia is a prolonged QTc. Cardiomyopathy or congestive heart failure may rarely result from prolonged hypocalcemia, cardiac arrhythmias [69]
	Increased neuromuscular excitability: tetany (when caused by respiratory alkalosis: hyperventilation-induced tetany), paresthesia, muscle spasms, and cramps, Chvostek’s sign, Trousseau’s sign, seizures [70,71]
	Central nervous system: ranging from generalized fatigue and depression to confusion, delirium or coma, calcification of the basal ganglia and other intracerebral calcifications [68]
Sodium (Na)	Mild symptoms: anorexia, nausea, vomiting, headache, muscle cramps [72]
	Moderate symptoms: muscle weakness, lethargy, confusion [73]
	Severe symptoms: seizures, altered consciousness, coma [73]
Potassium (K)	Muscular: muscle weakness, paralysis, muscle cramps and spasms, deep tendon reflexes [74,75]
	Cardiovascular: cardiac arrhythmias (e.g., premature atrial and ventricular complexes, ventricular fibrillation [76]
	Other symptoms: nausea, vomiting, constipation, fatigue, polyuria [74,77]
Phosphorous (P)	Cardiovascular system: hypophosphatemic cardiomyopathy, and arrhythmias [78,79]
	Muscle composition: total phosphorous and magnesium are abnormally low, whereas sodium, chloride, and calcium are abnormally elevated. Potassium content of skeletal muscle is low. The muscle weakness and osteomalacia can occur in severe chronic depletion of profound hypophosphatemia may be accompanied by rhabdomyolysis, especially in acute alcoholism [80]
	Respiratory insufficiency: hypoxia, respiratory acidosis, peripheral neuropathy, weakness, or muscle paralysis [81,82]
	Erythrocyte/leukocyte dysfunction/platelet disorders: hematologic disturbances of profound hypophosphatemia include hemolytic anemia, decreased release of oxygen from hemoglobin, as well as impaired leukocyte and platelet function. 50% reduction in chemotactic, phagocytic, bactericidal activity of granulocytes, 2,3-DPG in erythrocytes [81]
	Nervous system: neuromuscular disturbances, progressive encephalopathy, seizures, coma, and death, brain stem dysfunction, peripheral neuropathy, encephalitis [65]
	Metabolic acidosis: mobilization of bone mineral and hypercalciuria, an increase in intracellular pH [82]
Selenium (Se)	Cardiovascular: Keshan disease is congestive cardiomyopathy associated with heart failure, cardiac enlargement, electrocardiogram (ECG) abnormalities, gallop rhythm, and even cardiogenic shock [83]
	Endocrine System: of the 35 selenoproteins that have been identified so far, 3 of them are called Iodothyronine deiodinases, and they play a role in thyroid hormone metabolism. The thyroid contains the maximum concentration of selenium of all the organs in our bodies. One of the iodothyronine deiodinases converts inactive thyroxine to its active form. The second one is abundant in the central nervous system (CNS), brown fat and skeletal muscles and also plays a role in the activation of thyroid hormones. The third has a role in deactivating thyroid hormones [84]
	Immune System: glutathione peroxidase (G-Px) is a selenium-dependent enzyme that protects cell membranes and lipid-containing organelles from peroxidative damage by inhibition and destruction. It acts in combination with vitamin E to maintain the integrity of the cell membranes, participating in redox reactions with hydrogen peroxide-producing glutathione. Selenium deficiency exacerbates the redox by-product toxicity and oxidative damage to cell membranes [85]
	Musculoskeletal: Kashin–Beck disease is a disabling deformity of bones, cartilage, and joints leading to enlarged joints and restricted movements. Both Keshan disease and muscular syndrome have been described in patients on total parenteral nutrition (TPN) who did not have selenium added to their supplement. Symptoms described included intermittent myalgias and tenderness as well as eventual white fingernail beds [86]
	Neurological and psychiatric: studies have shown that selenium deprivation can lead to depressed mood and more hostile behavior. The turnover rate of some neurotransmitters has also been found to be affected by selenium deficiency. Selenium concentration in the brain of patients with Alzheimer’s disease was found in one study to be about 60% of that of controls [87,88]
	Reproductive System: selenium is essential for testosterone biosynthesis and the formation and development of normal spermatozoa. Testicular tissue contains large concentrations of selenium and is responsible for sperm quality and male fertility health [86]
Zinc (Zn)	Brain: neuropsychiatric disorders, neurosensory disorders, decreased nerve conduction, mental lethargy [89,90]
	Thymus: thymic atrophy [91]
	Skin: skin lesions, acrodermatitis, decreased wound healing [92]
	Reproductive system: hypogonadism [91]
Chromium (Cr)	Chromium insufficiency has been hypothesized to be a contributing factor to the development of type 2 diabetes and atherosclerosis [93]
	Psychiatric: an abrupt rise in anxiety levels, a sudden decrease in energy level, chronic fatigue, signs of muscle weakness, slow growth rate, mood swings [93]

**Table 3:** The normal range, and deficiency conditions of trace/macroelements in the serum of adults [128,129,130,131,132].

Trace/Macro Elements	The Reference Range	Minerals Deficiency Conditions	Trace/Macro Elements	The Reference Range	Minerals Deficiency Conditions
sodium	135–145 mEq/L (135–145 mmol/L)	Hyponatremia: serum concentration < 135 mEql/L Hypotonic hyponatremia: serum osmolality < 280 mOsmol/kg Hypertonic hyponatremia: serum osmolality > 295 mOsmol/kg Isotonic hyponatremia: serum osmolality 280–295 mOsmol/kg	calcium	9 to 10.5 mg/dL (2.2–2.6 mmol/L)	Total serum calcium concentration < 8.5 mg/dL (<2.12 mmol/L), or ionized (or free) calcium concentration < 4.65 mg/dL (<1.16 mmol/L)
potassium	3.5 to 5 mmol/L	serum level < 3.5 mEq/L	magnesium	1.8–3.0 mg/dL (0.8–1.2 mmol/L)	serum Mg < 1.6 mg/dl
chromium	−0.05–0.5 μg/L (1–10 μmole/L)	No data	selenium	less than 8 μg/dL	No data
zinc	84–159 μg/dL	No data	phosphorous	3.0–4.5 mg/dL (1.0–1.5 mmol/L)	serum phosphate < 2.5 mg/dL (0.81 mmol/L).
